# Epidemiological Evidence Supports the Role of Microbial Interactions in Polymicrobial UTI Infections Revealed by In Vitro Research

**DOI:** 10.3390/antibiotics14101028

**Published:** 2025-10-14

**Authors:** Gabriella Piatti, Alessandro Mannini, Alberto Vitale, Marco Bruzzone, Anna Maria Schito, Marcello Ceppi

**Affiliations:** 1 DISC—Department of Surgical Sciences and Integrated Diagnostics, University of Genoa, 16132 Genoa, Italy; amschito@unige.it; 2 Unit of Microbiology, IRCCS Ospedale Policlinico San Martino, 16132 Genoa, Italy; alberto.vitale@hsanmartino.it; 3 CNR-IRBIM—Institute for Marine Biological Resources and Biotechnology, 60125 Ancona, Italy; alessandro.mannini@irbim.cnr.it; 4 Unit of Epidemiology, IRCCS Ospedale Policlinico San Martino, 16132 Genoa, Italy; marco.bruzzone@hsanmartino.it (M.B.); ceppimar@gmail.com (M.C.)

**Keywords:** UTI, epidemiology, polymicrobial infections, microbial interaction, microbial synergy, uncompromised host, compromised host

## Abstract

**Background**: Molecular techniques for microbial identification have highlighted the relevance of polymicrobial infections in humans, such as those affecting the urinary tract. Although in vitro investigations have demonstrated connections between co-infections and microbial interaction, their role is unclear in clinics, given the overlap with host conditions. **Objective**: We aimed to separate the roles of organisms and patient conditions in human polymicrobial urinary samples by performing a relevant epidemiological analysis. **Methods**: We analyzed retrospective results from urine cultures performed during one year in a 1200 beds Italian hospital. Patients were grouped as uncompromised and compromised and positive urine cultures were grouped as monomicrobial and polymicrobial. We assessed associations between single microorganisms and the groups of positive samples and between single microorganisms and the group of patients through a multivariate logistic regression model, adjusting by the confounding effect of seven variables. **Results**: We enrolled 24,067 urine samples, among which 7208 were positive, 75% monomicrobial and 25% polymicrobial. We found that the polymicrobial samples had a microbial scenario wider than the monomicrobial ones and the organisms most sampled had the highest number of different pairwise associations. Certain organisms shown having absolute numerical advantages in the polymicrobial urine cultures with respect to the monomicrobial ones, independently of host’s conditions. **Conclusions**: The numerical advantage shown by certain organisms in polymicrobial urine samples over monomicrobial samples supports the hypothesis of microbial synergies favouring the occurrence of certain co-infections.

## 1. Introduction

Urinary tract infections (UTIs) are the most frequent bacterial diseases in humans and the predominant affection in the tract [1]. Complicated UTIs, whose incidence, though considerable, is overshadowed by infections in other organs, are those most occurring in the hospital setting. Together with the comorbidity of patients, referred to as inpatients or compromised hosts, the hospital setting represents the main risk of infection [2]. Uncomplicated UTIs occur in community affecting otherwise healthy individuals, referred to as outpatients or uncompromised hosts. Uncomplicated UTIs, especially in industrialized countries, stand out for their much higher incidence than infections in other parts of the human body [3]. All ascendant UTIs, the most frequent being both among compromised and healthy subjects, are of endogenous origin due to opportunistic microorganisms, which are part of the native intestinal microbiota [4]. Nearly no microorganisms involved in ascendant human UTIs are pathogenic [5]. This is an exception among the infections in other organs, none of which in healthy individuals are exclusively due to commensal bacteria and fungi [6,7]. Absolute pathogenic bacteria such as *Salmonella typhi* and *Brucella melitensis* reach the urinary tract and cause UTIs through the haematogenous route [8,9].

In the past two decades, thanks to the extraordinary development of molecular techniques and culture-independent identifications, polymicrobial infections emerged as more numerous and clinically important than previously thought [10,11,12].

Basic research has led to an understanding of the mechanisms of microbial interactions promoting selective advantages, whether dependent on or independent of the environment, and resulting in mutual presence at infection sites [13]. Interaction between different microbial species can enable more effective colonization, by improving conditions that favour mutual growth or providing essential nutrients, assessed among catheter-associated urinary tract infection (CAUTI) through the biofilm formation [13,14]. Competition is the phenomenon that results from interaction between different microorganisms and that is most easily understood and most widely studied [15]. The phenomenon of microbial synergy, less supported in its existence by basic research, is nevertheless well represented in studies addressing the modulation of host immune response, such as modulation by the toll-like receptor (TLR) signalling system [16,17,18]. Two properties make it plausible to consider the involvement of TLRs in synergic microbial interaction: the different and opposite pro-inflammatory and anti-inflammatory functions of co-receptors, such as the well-studied CD14, co-receptor of TLR4 [19], and the common signalling and adaptor molecules that enable interaction between different stimuli, activators and suppressors, from different microbial species [20].

Apart from the comprehensive work of Nye et al. [21], statistics on human UTIs have given little information on the microbial benefits, or even dams, related to the interaction between different organisms. Animal polymicrobial models of disease and statistics on human UTIs have highlighted the responsibility of host fragility in the onset of mixed forms [22] and revealed the severity of and influence on the healing process and overall clinical course [23,24,25].

In this study, we retrospectively analyzed positive monomicrobial and polymicrobial urinary cultures from both healthy and compromised patients. The epidemiological evidence allowed us to distinguish the role of patients and the role of individual organisms in microbial urinary positivity, which is not sufficient to define the infectious disease but is necessary for the first crucial step.

## 2. Results

### 2.1. Patients and Urinary Samples

**Figure 1:**
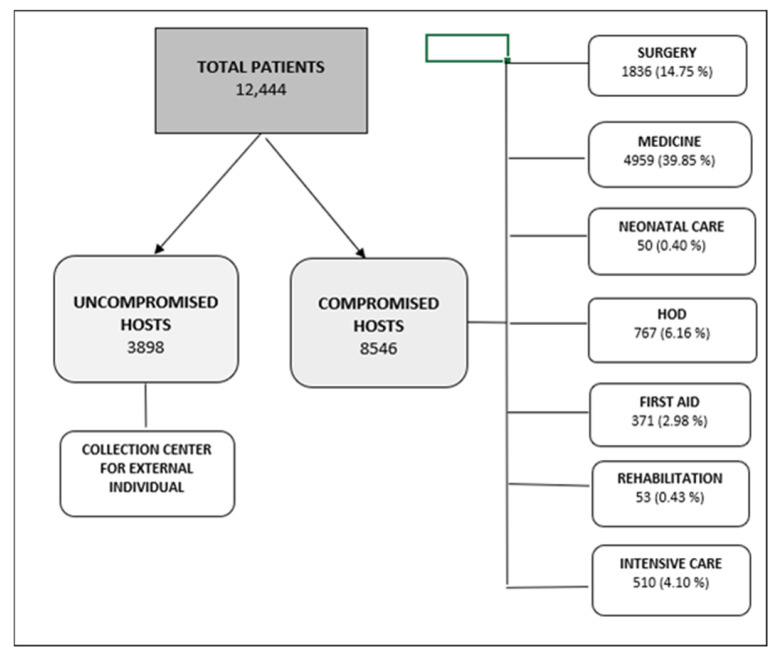
Flowchart showing provenance of uncompromised hosts and ward provenance of compromised hosts enrolled during the study. HOD, Haemato-Oncological Diseases.

The flowchart depicted in Figure 1 shows the subjects enrolled, i.e., uncompromised hosts, compromised hosts, and the distribution of the latter (i.e., inpatients, admitted in single groups of wards of hospitalization, and outpatients, referred to the relevant outpatient clinics), who gave urinary samples, which are the object of this study.

Table 1 shows the demographic characteristics and prevalence of 12,444 enrolled subjects from whom urine samples were obtained, uncompromised hosts (3898) and compromised hosts (18,409). Female gender was more frequent among uncompromised than among compromised hosts (*p* < 0.001), age under 65 years was more frequent among compromised hosts (*p* < 0.001).

Table 1 also shows the microbiological results from 24,067 urinary cultures, negative and positive, the latter listed as monomicrobial, the main part, and polymicrobial. Positive cultures from overall, uncompromised and compromised hosts were 31%, 29% and 32%, respectively. Female gender was more frequent among uncompromised than among compromised hosts having giving total, negative, positive, monomicrobial and polymicrobial urine samples (*p* < 0.001). Age under 65 years was more frequent among compromised hosts having giving total, negative, positive, monomicrobial and polymicrobial urine samples (*p* < 0.001). Polymicrobial urine samples were more frequent among compromised hosts than among uncompromised hosts (*p* = 0.036). No urine samples showed more than two microorganisms at CFU ≥ 1 × 10^5^/mL.

### 2.2. Microbial Genera and Species According to Types of Positive Cultures and Groups of Patients

Table 2 shows number and percentage of different microbial genera and species according to types of positive urinary cultures (monomicrobial or polymicrobial) and to groups of patients (healthy or compromised). Significant differences emerged from both evaluations. Mixed samples (more frequent among the compromised hosts over the uncompromised ones, see Table 1) showed greater percentage of different organisms than the monomicrobial ones (more clearly in case of genera, among uncompromised hosts, *p* < 0.001, than among the compromised hosts, *p* = 0.005). Samples from uncompromised hosts showed greater percentage of different organisms than from compromised hosts (more clearly in case of genera, among mixed cultures, *p* < 0.001, than among monomicrobial cultures, *p* = 0.004).

The global number, genera and species of isolates from all positive urine cultures are depicted in Table S1. Samples were grouped in monomicrobial samples from compromised hosts (4168), monomicrobial samples from uncompromised hosts (1310), polymicrobial samples from compromised hosts (1424) and polymicrobial samples from uncompromised hosts (378).

### 2.3. Urinary Microbial Positivity, Frequency of Species Present and Number of Different Paired Organisms

Table 3 shows the number and percentage of positive urinary cultures from which individual organisms were isolated, and the number of different organisms associated with each of them, up to five, in different mixed samples. The Table shows that the most frequently sampled organisms had the largest number of different pairwise associations. This finding suggests that the larger is the pattern of association the greater the growth advantage for the coupled organisms. The first ten species isolated were, in decreasing order, *Escherichia coli*, *Enterococcus faecalis*, *Klebsiella pneumoniae*, *Enterococcus faecium*, *Proteus mirabilis*, *Pseudomonas aeruginosa*, *Candida glabrata*, *E. Species, Morganella morganii*, *Citrobacter freundii*.

### 2.4. Positive Pairwise Microbial Associations

We considered microorganisms present in at least five urinary samples and found very few significant positive microbial associations. Among the compromised hosts, two *Candida* species (*C. albicans*, *Candida glabrata*) were variously associated with each other and with *E. faecium* (ORs ranging from 2.67 to 8.08). Among the uncompromised hosts, we found one positive association, between *S. haemolyticus* and *E. faecalis* (OR = 5.25) (Table 4).

### 2.5. Associations Between Individual Microorganisms and Groups of Patients

**Figure 2:**
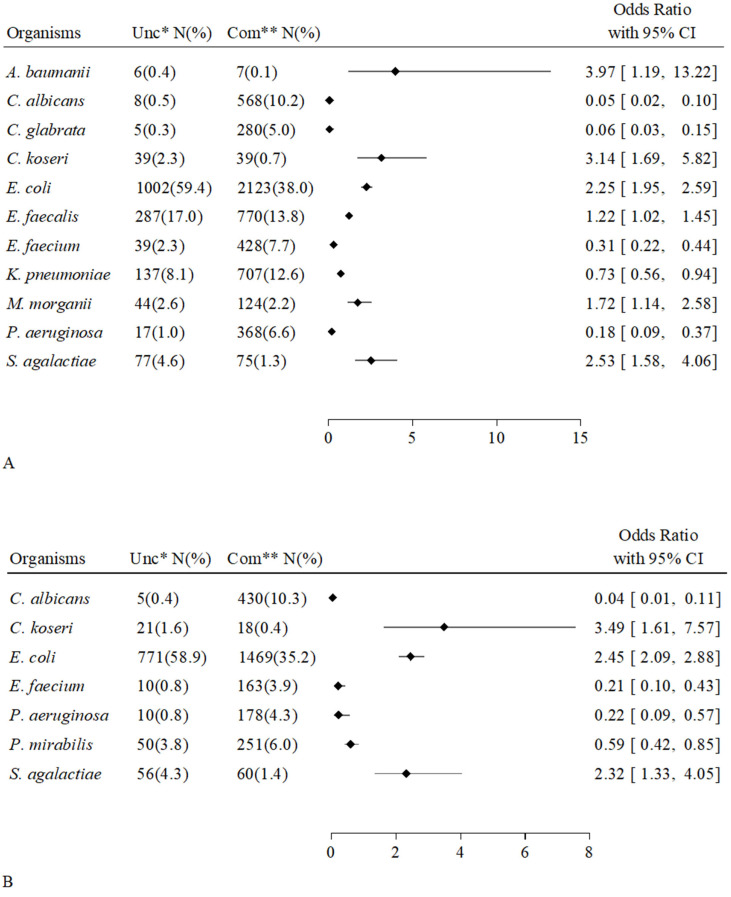

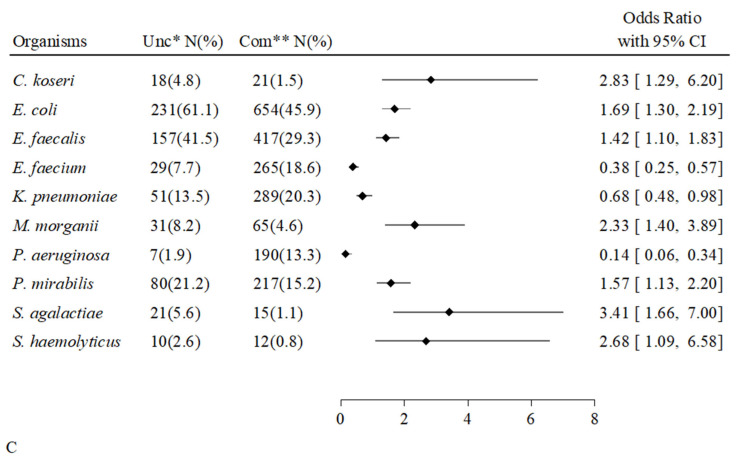
Forest plot showing a list of microorganisms associated with uncompromised hosts (OR > 1) versus the compromised ones (OR < 1), grouped as all, monomicrobial, and polymicrobial urinary cultures. Odds Ratio and its 95% Confidence Interval (95% CI) were estimated through a logistic model by adjusting for gender and age, hospitalization ward, immunosuppressive treatment, infection/s or colonization in other sites by the same microorganism. (**A**), All the samples (also adjusted whether urinary samples were polymicrobial or monomicrobial); (**B**), Monomicrobial samples; (**C**), Polymicrobial samples. * Uncompromised hosts; ** Compromised hosts.

We considered all microorganisms sampled in at least five urine cultures. Figure 2 shows the microbial species associated with individual patient groups, grouped in all positive cultures, monomicrobial cultures, and polymicrobial cultures. Among overall positive cultures (A), eleven microorganisms were significantly associated with one patient group, i.e., six with uncompromised hosts and five with compromised hosts. Among monomicrobial cultures, three microorganisms were associated with uncompromised hosts (*Citrobacter koseri*, *E. coli*, *S. agalactiae*) and four microorganisms were associated with compromised hosts (*Candida albicans*, *E. faecium*, *P. aeruginosa*, *P. mirabilis*) (B). Among polymicrobial cultures, seven bacterial species were associated with uncompromised hosts, and three bacterial species were associated with compromised hosts (C). The scenario of uncompromised host-organism associations expanded in polymicrobial forms with the acquisition of four bacteria (*E. faecalis*, *M. morganii*, *P. mirabilis*, *S. haemolyticus*). The scenario of uncompromised host-organism associations was reduced in mixed cultures, retaining *E. faecium* and *P. aeruginosa*, losing *C. albicans* and *P. mirabilis*, and acquiring *K. pneumoniae*.

### 2.6. Associations Between Individual Microorganisms and Types of Positive Cultures

**Figure 3:**
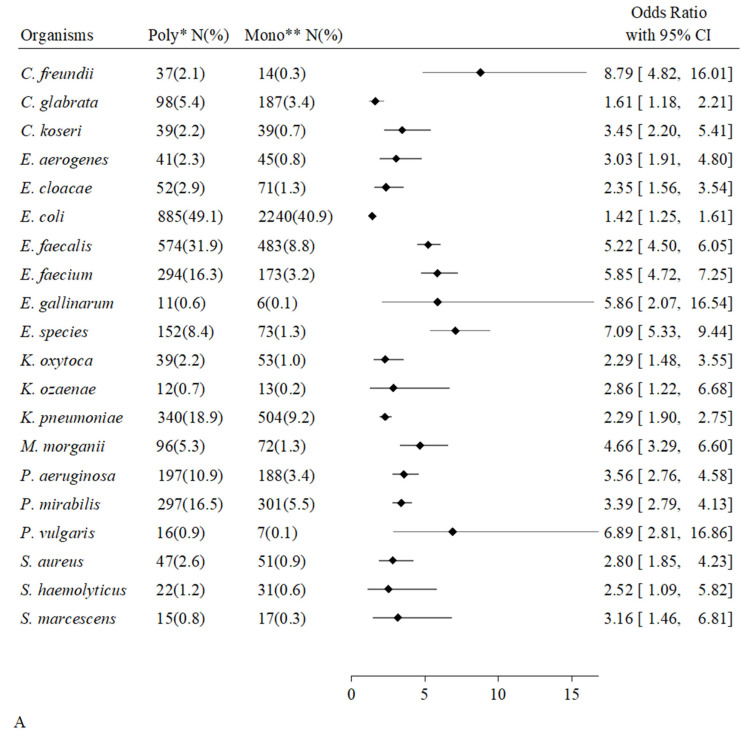

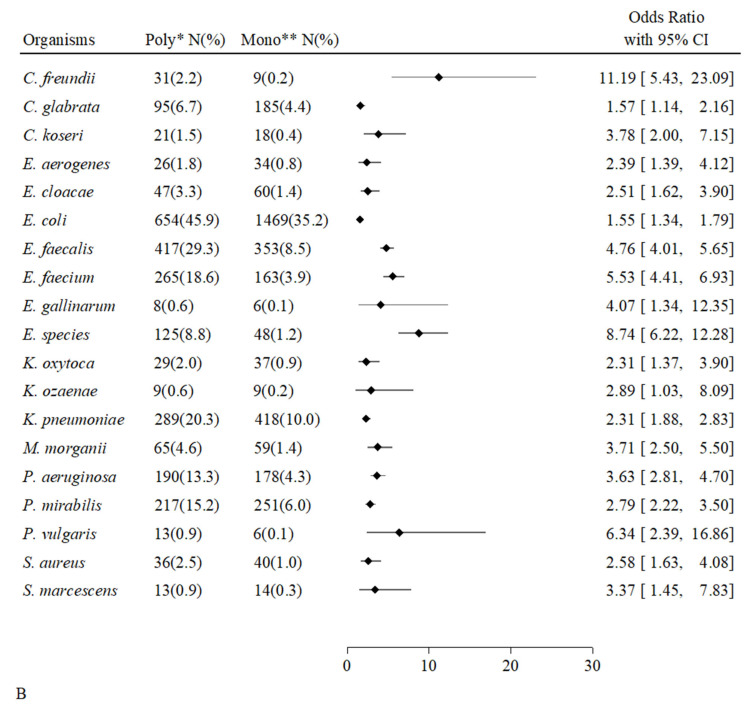

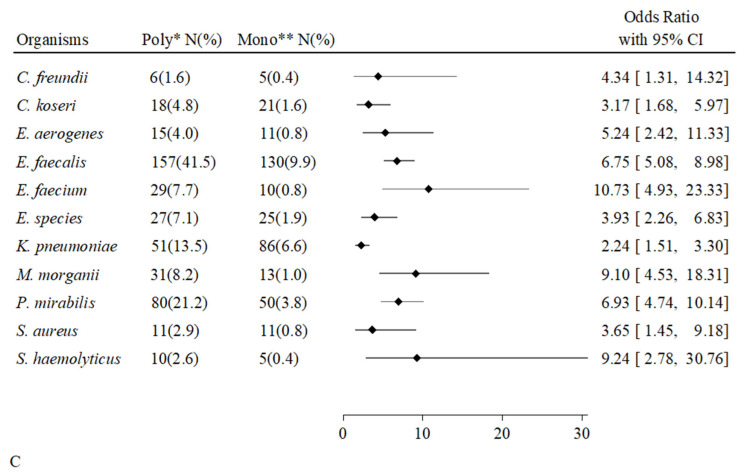
Forest plot shows a list of microorganisms associated with the polymicrobial urinary cultures, grouped in origin from all, compromised hosts, and uncompromised hosts. Odds Ratio and its 95% Confidence Interval (95% CI) were estimated through a logistic model by adjusting for gender and age, hospitalization ward, immunosuppressive treatment, infection/s or colonization in other sites by the same microorganism. (**A**), All hosts (also adjusted whether hosts were compromised or uncompromised); (**B**), Compromised hosts; (**C**), Uncompromised hosts. * Polymicrobial samples; ** Monomicrobial samples.

We considered all microorganisms sampled in at least five urine cultures. Figure 3 shows the microbial species associated with polymicrobial cultures, grouped according to the origin of the samples from all hosts, compromised and uncompromised. Among samples from all patients, twenty microbial species were associated with polymicrobial cultures (A). Eight microbial species were isolated from a greater absolute number of polymicrobial cultures than monomicrobial cultures (*C. freundii*, *E. faecalis*, *E. faecium*, *Enterococcus gallinarum*, *Enterococcus species*, *M. morganii*, *P. aeruginosa*, *Proteus vulgaris*). Among compromised hosts, we found the same nineteen species associated with polymicrobial cultures and the same species, plus *C. koseri*, more numerous in mixed cultures (B). Among uncompromised hosts, eleven bacterial species were associated with mixed cultures, and almost all of them, i.e., eight bacterial species, were more numerous in mixed cultures (C). *C. freundii*, *E. faecalis*, *E. faecium*, *E. species*, and *M. morganii* were isolated in greater absolute numbers from polymicrobial cultures and were all shared by uncompromised and compromised hosts.

**Figure 4:**
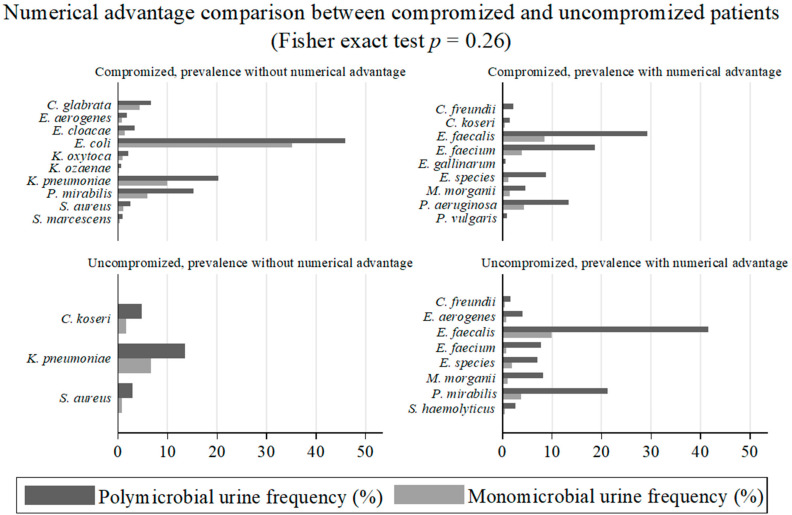
Number and species of microorganisms with advantage or not among the two groups of patients. *p*- value significant when < 0.005.

Figure 4 shows the prevalent organisms in mixed cultures and those that were isolated in greater absolute numbers (with numerical advantage) from polymicrobial cultures or not, based on patients’ groups. The difference in the numerical advantage of the organisms isolated from polymicrobial urinary samples between the two host groups was not statistically significant when performing Fisher’s exact test (*p* = 0.26). Therefore, we can affirm that “a numerical advantage from polymicrobial condition” for certain microorganisms did not appear to be related to host conditions.

### 2.7. Associations of Individual Microorganisms According to Groups of Patients and Types of Positive Urinary Samples

Table 5 shows that the variables so far treated separately influenced each other and generated different ORs depending on the levels they defined jointly. It emerged that five bacterial species, *E. coli, E. species, M. morganii, P. mirabilis* and *S. haemolyticus*, had very different ORs in the joint levels of the two interacting variables. Having set as reference level the probability of microbial presence occurring in the monomicrobial urine of compromised patients (OR of 1), *E. coli*, and to a lesser extent *E. species*, showed greater frequency in monomicrobial urine samples of uncompromised patients. Polymicrobial urine samples did not seem to give an interesting advantage to *E. coli* presence, giving instead to *E. species*, whose frequency was higher precisely in polymicrobial forms and more in compromised hosts. In contrast, *M. morganii*, *P. mirabilis* and *S. haemolyticus* were less frequent in uncompromised than in the compromised hosts among monomicrobial urine, while among those polymicrobial they exceed the baseline level, and much in the uncompromised hosts. Finding microorganisms associated with compromised host among urine monomicrobial and others associated with uncompromised host among the polymicrobial did suggest that specific microbes might favour the occurrence of mixed infections more than poor host conditions.

## 3. Discussion

The aim of this study was to evaluate the influence of host conditions, described by clinics, and the influence of microorganisms, described by basic research, in urinary polymicrobial co-infections in humans.

With the advent of culture-independent identifications, the increasing recognition of mixed infection in the human bloodstream has enabled the diagnosis of mixed forms even at other sites, including the urinary tract [11,12,26].

UTIs offer extraordinary opportunities for investigations, given the large number of complicated and uncomplicated cases and the very high frequency of monomicrobial and polymicrobial forms, the latter prevalent among compromised hosts and significant among those uncompromised [2,27]. UTIs also deserve particular interest given the notable, nearly exclusive, presence of endogenous, not strictly pathogenic organisms in the non-native and sterile tracts as cause of infection [28].

While planning our study, we kept into account the following considerations: it is known that the more the host is compromised the lower the microbial virulence required to break the host–parasite balance and cause infection [29]; consequently, the role of host fragility and microbial virulence in polymicrobial infections could obscure each other; basic mechanistic studies showed that some co-infections result from the mutual interaction between different microbes and their virulence [15,30,31,32,33,34].

Our demographic findings are consistent with most of the findings reported in the epidemiological literature on UTIs: prevalence of female gender among healthy subjects, prevalence of age under 65 among compromised hosts, organisms most frequently sampled from urine samples, and prevalence of polymicrobial forms among compromised hosts compared to non-compromised hosts [3,22,35].

To our knowledge, the following results from our analyses have never been reported before. We found that the microbial scenario of overall samples by uncompromised hosts was broadest, as well as, more predictably, the scenario of overall polymicrobial samples. Furthermore, the organisms that exhibited the greatest number of diverse associations were those isolated most frequently, indirectly suggesting some sort of advantage for those organisms that had achieved the broadest microbial interactions. We take as examples *E. coli* and *E. faecalis*, which both met these combined characteristics, representing, respectively, 42% and 14% (the first and second in frequency terms) of all organisms isolated in urinary samples, and globally paired with 45 and 44 different species (Table 3). This study reveals in fact several multiple, i.e., common microbial associations, which we do not consider as random anyway, but reminiscent of the common adaptor molecules of TLRs, such as MyD88, whereby stimuli from different microbial species lead to a common result, and the multifaced role of CD14 co-receptor of TLR4. Several studies describe the significant role that direct or indirect inhibitors of the TLR-dependent pro-inflammatory pathway plays in infections and inflammatory conditions in humans [36,37,38,39]. In vitro stimulation of a bladder cell line with the non-pathogenic strain *E. coli* K-12 causes TLR4 activation and inflammatory cytokine response (leading to phagocyte recruitment), while stimulation with uropathogenic *E. coli* (UPEC) reduces cytokine production and activation of immune surveillance, even during co-infection with activators [40,41].

Another finding concerns the close and specific association between eleven microbes and individual host groups [Figure 2]. Interestingly, the number of organisms associated with uncompromised subjects increased from monomicrobial to mixed samples: urine cultures from healthy individuals acquired associations with *E. faecalis*, *M. morganii*, *S. haemolyticus*, and *P. mirabilis*, while maintaining those with *C. koseri*, *E. coli*, and *S. agalactiae*. *P. mirabilis* was the only species whose association was shared by both host groups: compromised hosts among the monomicrobial samples and uncompromised hosts among the polymicrobial samples.

The key finding concerns the apparent role of microbial interaction in polymicrobial infections, which emerged from evidence of a numerical advantage that some organisms gained in mixed urinary growth. These organisms, associated with polymicrobial samples, were isolated in greater absolute numbers than in monomicrobial samples [Figure 3]. Although partially different in species type, these were numerically equal in the two host groups (*p* = 0.26). Among the uncompromised hosts, the organisms that “benefited” in mixed forms were *C. freundii*, *E. aerogenes*, *E. faecium*, *E. species*, *E. faecalis*, *M. morganii*, *P. mirabilis*, and *S. haemolyticus*. The latter four species were the same as those “recruited” by the group of healthy hosts from monomicrobial forms to polymicrobial forms, described in the previous paragraph and depicted in Figure 2. Individual species associated with polymicrobial infections without a numerical advantage, i.e., apparently by chance, were definitely more numerous among compromised hosts than among uncompromised hosts, thus explaining the prevalence of polymicrobial urine samples in this patient group compared to healthy individuals.

Recalling the *E. coli*-*E. faecalis* couple, *E. coli* was associated, among compromised hosts, with polymicrobial urine samples without a numerical advantage. Conversely, *E. faecalis* was associated, in both patient groups, with polymicrobial samples with a clear numerical advantage. We hypothesized that organisms like *E. coli* may non-specifically but non-randomly share their ability to enter the urinary tract with different species, which, in turn, may benefit non-specifically and non-randomly from different interactions, leaving both organisms little room for specific associations. This pattern reflects the dynamics of monomicrobial UTIs, of which UPEC strains are the primary cause and paradigm, both among uncompromised and compromised hosts, while still allowing room for other organisms less equipped to invade or persist in the urinary tract in a seemingly random manner [3].

In their work, Nye et al. found thirteen positive (such as between *E. coli* and *E. faecalis*) and thirteen negatives pairwise associations more often than expected by chance among catheter-associated urinary tract infections [21]. The authors supported and explained their findings with in vitro co-culture, where the presence of *E. coli* significantly increased the growth of *E. faecalis*. We believe that the conclusions of our work and those of Nye et al. are not mutually exclusive, but rather complement each other, sharing evidence of a microbial advantage, for growing or to be present in human urinary tract, and may result from activity of one organism engaging another.

We can state that both host fragility and specific microorganisms appear to play a significant role in the onset of co-infections and that microbial capabilities appear to be in line with what was observed in vitro in the studies we referred to [40,41].

We acknowledge two structural limitations to our work, both due to the retrospective nature. First, we did not indicate how many patients had urinary catheter because of the lack of such data in the ward data repository. However, we believe this limit has an attenuated impact on interpretation of data because both indwelling and extemporaneous urinary catheters, the great risk factor for polymicrobial UTIs, only concerns compromised hosts, representing a great risk factor as well. Second, our diagnostics identified nineteen bacteria as *Enterococcus species*, affecting statistic. Another structural limit is the lack of epidemiology of the antibiotic susceptibility phenotype of involved microorganisms. We decided not to report it precisely because of its extent, including issues related to the complexity of clinical management and to microbial fitness [13,42].

Despite the limitations of our work, the results highlighted the role of individual microorganisms and interactions in co-infections, regardless of host conditions, whose importance is nevertheless evident. There is a clear need to better understand the dynamics in order to improve the therapeutic management of infections and prevent the onset of life-threatening forms.

## 4. Methods

### 4.1. Database

This was a retrospective study from reporting and record software (TD-Synergy MultiLab, SIEMENS) in use at our institution, a 1200-bed Italian hospital (Genoa). The cohort consisted of patients hospitalized and subjects in the community from the same regional area, categorized as compromised and uncompromised hosts. Data concerned results from urine cultures performed in the hospital Microbiology laboratory. The study period was from January to December 2017.

### 4.2. Samples Collection and Microbial Cultures

Urinary specimens were cultured for 12 months in the Unit of Microbiology, IRCCS San Martino Hospital, Genoa (Italy) following the standardized protocols for routine collection of midstream clean catch urine and for collection from urinary catheter. The samples arrived at laboratory within one hour from collection and were inserted into the automated device ((Alfred Sidecar semina, Alifax), Padova, Italy) for screening positive samples. Urine signed as positive was seeded on Columbia and Crom-det agar (Liofilmchemsrl, Teramo, Italy) and incubated in aerobiosis for 24 h. Identification of bacteria and yeasts colonies grown on solid media was performed with the MALDI-TOF system.

### 4.3. Patients and Urinary Samples and Definitions

Among enrolled subjects, we separated the group of compromised hosts from the group of uncompromised hosts. The first group included patients admitted to hospital for conditions other than urinary tract infections and individuals from the community with underlying conditions for which they were treated in relevant outpatient clinics. Among this group we considered three different subgroups: (1) subgroup according to types of admission ward and the relevant outpatient clinic, (2) subgroup according to immunosuppressive therapy administered or not, (3) subgroup of patients with positive urine for microorganisms present or not present at other sites of infection or colonization, from the month before to the month after the urinary culture. The second group included outpatients healthy except by suspected UTI, afferent to the CCOI (Collection Centre for External Individuals).

Positive urine samples enrolled for the study were those where microorganisms were present at CFU (Colony Forming Units) ≥1 × 10^5^/mL. Positive samples were categorized as monomicrobial and polymicrobial, depending on whether one or at least two microorganisms were equally isolated at CFU ≥1 × 10^5^/mL. Samples where one or more microorganisms were present at CFU <1 × 10^5^/mL were excluded. Urine cultures without growth after the screening were defined as negative.

### 4.4. Statistical Analysis

The connection between demographic variables and patient healthy condition, uncompromised and compromised, the type of positive results (monomicrobial and polymicrobial), as between number of microorganisms’ type (genera and species) found in the entire study, was tested with the chi-square statistic or Fisher test when it is appropriate.

The association of each microorganism with the type of urinary result and with patient conditions was checked through a multivariate logistic regression model, adjusting by the confounding effect of gender, age, hospitalization ward, immunosuppressive treatment, infection/s or colonization other sites by the same microorganism/s and, when required, the origin of patients and type of positive result [43]. The Odds Ratio (OR) provided by this model estimates the risk of microbial presence linked to a specific factor. The same method was applied for evaluating the link between each microorganism and the other ones. Since repeated examinations on the same patient were collected, representing a cluster of correlated observations, the standard errors of ORs were estimated considering the intra-patient correlation. The Wald test was applied to check the statistical interaction between the source of patients and the type of urinary microbial presence. STATA software version 17 was used for all statistical analyses [44].

## Figures and Tables

**Table 1:** (**A**) Demographic characteristics of patients from whom urinary samples were obtained during the study. (**B**) Microbiological results of urine cultures according to the groups of hosts.

	Overall Hosts		Uncompromised Hosts		Compromised Hosts		*p*-Value
A							
	N	%	N	%	N	%	
Total hosts	12,444		3898		8546		
females	6417	(52)	2366	(61)	4051	(47)	<0.001
males	6027	(48)	1532	(39)	4495	(53)	
≥65 years	5301	(43)	1892	(49)	3409	(40)	<0.001
<65 years	7143	(57)	2006	(51)	5137	(60)	
B							
Total cultures	24,067		5658		18,409		
negative cultures	16,787		3970		12,817		
from							
total hosts	10,004	(69)	3107	(71) *	6897	(68) *	0.001 *
females	4730	(47)	1734	(56)	2996	(43)	<0.001
males	5274	(53)	1373	(44)	3901	(57)	
≥65 years	4583	(46)	1577	(51)	3006	(44)	<0.001
<65 years	5421	(54)	1530	(49)	3891	(56)	
positive cultures	7280		1688		5592		
from							
total hosts	4431	(31) *	1250	(29) *	3181	(32) *	
females	2831	(64)	968	(77)	1863	(59)	<0.001
males	1600	(36)	282	(23)	1318	(41)	
≥65 years	1431	(32)	496	(40)	935	(29)	<0.001
<65 years	3000	(68)	754	(60)	2246	(71)	
monomicrobial cultures	5478		1310		4168		
from							
total hosts	3609	(72)	1010	(75) **	2599	(72) **	
females	2279	(63)	781	(77)	1498	(58)	<0.001
males	1330	(37)	229	(23)	1101	(42)	
≥65 years	1184	(33)	403	(40)	781	(30)	<0.001
<65 years	2425	(67)	607	(60)	1818	(70)	
polymicrobial cultures	1802		378		1424		
from							
total hosts	1377	(28) **	344	(25) **	1033	(28) **	0.036 **
females	893	(65)	268	(78)	625	(61)	<0.001
males	484	(35)	76	(22)	408	(39)	
≥65 years	400	(29)	128	(37)	272	(26)	<0.001
<65 years	977	(71)	216	(63)	761	(74)	

*p*-values, chi-squared. * *p*-values, chi-squared from comparison between frequencies of negative and positive urine among uncompromised and compromised hosts. ** *p*-values, chi-squared from comparison between frequencies of polymicrobial and monomicrobial urine among uncompromised and compromised hosts.

**Table 2:** Number and percentage of different microbial genera and species according to groups of hosts and types of positive urine cultures.

	Positive Urine Cultures		
	polymicrobial N (%)	monomicrobial N (%)	*p*-value ^1^
**Microbial genera**			
**from Hosts**			
Uncompromised	17 (4.5)	20 (1.5)	<0.001
Compromised	21 (1.5)	28 (0.7)	0.005
*p*-value ^2^	<0.001	0.004	
**Microbial species**			
**from Hosts**			
Uncompromised	41 (10.8)	42 (3.2)	<0.001
Compromised	61 (4.3)	69 (1.7)	<0.001
*p*-value ^2^	<0.001	<0.001	

*p*-value ^1^, chi-squared from comparison between polymicrobial and monomicrobial urine within the type of patient. *p*-value ^2^, chi-squared from comparisons between types of patients within polymicrobial or monomicrobial urine.

**Table 3:** Individual microbial species, number of the relevant positive urine cultures and number of different 2nd organisms isolated from the same sample. The list covers the organisms having up to five different second organisms.

Microbial Species	Isolated from Samples N	%	Coupled with 2nd Species N	Microbial Species	Isolated from Samples N	%	Coupled with 2nd Species N
*E. coli*	3125	42.93	45	*K. oxytoca*	92	1.26	10
*E. faecalis*	1057	14.52	44	*S. maltophilia*	20	0.27	9
*E. faecium*	467	6.41	31	*C. parapsilosis*	94	1.29	9
*K. pneumoniae*	844	11.59	31	*S. haemolyticus*	53	0.73	9
*P. mirabilis*	598	8.21	29	*S. marcescens*	32	0.44	9
*P. aeruginosa*	385	5.29	27	*P. putida*	10	0.14	8
*C. glabrata*	285	3.91	19	*P. stuartii*	18	0.25	7
*E. species*	225	3.09	19	*A. baumanii*	13	0.18	7
*M. morganii*	168	2.31	19	*P. vulgaris*	23	0.32	6
*C. freundii*	51	0.70	19	*S. fonticola*	8	0.11	5
*C. albicans*	576	7.91	17	*K. ozaenae*	25	0.34	5
*E. cloacae*	123	1.69	14	*S. hominis*	13	0.18	5
*S. agalactiae*	152	2.09	12	*S. epidermidis*	45	0.62	5
*E. aerogenes*	86	1.18	11	*C. tropicalis*	44	0.60	5
*C. koseri*	78	1.07	11	*P. rettgeri*	11	0.15	5
*S. aureus*	98	1.35	11	*E. avium*	6	0.08	5

**Table 4:** Microorganisms positively associated among polymicrobial urine cultures.

First Organism from Hosts	Second Organism	First AND Second Organisms	First Organism Alone	Odds Ratio (95% CI)
All				
*C. albicans*	*C. glabrata*	30 (21.3)	68 (4.1)	4.29 (2.48–7.40)
*C. albicans*	*C. tropicalis*	7 (5.0)	8 (0.5)	7.93 (2.92–21.5)
*C. albicans*	*E. faecium*	61 (43.3)	233 (14.0)	4.09 (2.59–6.46)
*C. glabrata*	*E. faecium*	34 (34.7)	260 (15.3)	2.62 (1.48–4.64)
Compromised				
*C. albicans*	*C. glabrata*	30 (21.7)	65 (5.1)	4.24 (2.45–7.33)
*C. albicans*	*C. tropicalis*	7 (5.1)	6 (0.5)	8.08 (2.86–22.82)
*C. albicans*	*E. faecium*	61 (44.2)	204 (15.9)	4.19 (2.62–6.68)
*C. glabrata*	*E. faecium*	34 (35.8)	231 (17.4)	2.67 (1.48–4.79)
Uncompromised				
*S. haemolyticus*	*E. faecalis*	8 (80.0)	149 (40.5)	5.25 (1.09–25.16)

Odds Ratio and its 95% Confidence Interval (95% CI) were estimated through a logistic model by adjusting for gender, age, to types of wards of admission, immunosuppressive therapy and infection in other sites or colonization by the same organism/s.

**Table 5:** Microorganisms for which a significant interaction effect occurs between types of positive urine samples and groups of patients.

	Monomicrobial Samples		Polymicrobial Samples	
	Compromised Hosts	Uncompromised Hosts	Compromised Hosts	Uncompromised Hosts
	ref	OR (95%CI)	OR (95%CI)	OR (95%CI)
*E. coli*	1	2.44 (2.08–2.86)	1.56 (1.35–1.81)	2.67 (2.11–3.38)
*E. species*	1	1.48 (0.89–2.44)	8.43 (5.99–11.86)	5.9 (3.59–9.7)
*M. morganii*	1	0.99 (0.51–1.91)	3.63 (2.44–5.39)	9.03 (5.5–14.82)
*P. mirabilis*	1	0.62 (0.44–0.88)	2.87 (2.29–3.6)	4.23 (3.05–5.86)
*S. haemolyticus*	1	0.89 (0.31–2.51)	1.55 (0.57–4.23)	6.99 (3.21–15.21)

## Data Availability

The data that supports the findings of this study are available from the corresponding author upon request.

## Supplementary Materials

The following supporting information can be downloaded at: https://www.mdpi.com/article/10.3390/antibiotics14101028/s1, Table S1: Overall microbial genera and species isolated from urinary cultures.
