# Supplementary material for: Epidemiological Evidence Supports the Role of Microbial Interactions in Polymicrobial UTI Infections Revealed by In Vitro Research
Source: Antibiotics (Basel). 2025 Oct 14;14(10):1028. doi: 10.3390/antibiotics14101028 (PMC12561107; doi:10.3390/antibiotics14101028)
Supplement: Supplementary file 1 [file antibiotics-14-01028-s001.zip › antibiotics-3813055-supplementary.pdf]

Table S1. Overall microbial genera and species isolated from urinary cultures

**Monomicrobial cultures from Inpatients total 4168**		**Monomicrobial cultures From Outpatients total 1310**		**Polymicrobial cultures From Inpatients total 1424**		**Polymicrobial cultures From Outpatients total 378**	
**GENERA 28**	**SPECIES 69**	**GENERA 20**	**SPECIES 42**	**GENERA 21**	**SPECIES 61**	**GENERE 17**	**SPECIE 41**
Acinetobacter	*baumanii*	*Acinetobacter*	*baumani*	*Acinetobacter*	*baumani*	*Acinetobacter*	*baumanii*
	lwoffi		*junii*		*ursingi*		*lwoffi*
Achromobacter	*xylosoxidans*		*lwoffi*	*Achromobacter*	*xylosoxidans*	*Aeromonas*	*spp*
Aerococcus	*spp*	*Aerococcus*	*spp*	*Aerococcus*	*spp*	*Candida*	*albicans*
Bacillus	*spp*	*Candida*	*albicans*	*Aeromonas*	*spp*		*glabrata*
Candida	*albicans*		*glabrata*	*Candida*	*albicans*		*tropicalis*
	dubliniensis		*tropicalis*		*glabrata*	*Citrobacter*	*amalonaticus*
	glabrata	*Citrobacter*	*amalonaticus*		*kefir*		*freundii*
	kefir		*freundii*		*parapsilosis*		*koseri*
	norvegensis		*koseri*		*krusei*		*stuarti*
	parapsilosis		*yungae*		*norvegiensis*		*yungae*
	tropicalis	*Enterobacter*	*aerogenes*		*guillermondii*	*Corynebacterium*	*striatum*
	utilis		*cloacae*		*utilis*	*Enterobacter*	*aerogenes*
Citrobacter	*braaki*	*Enterococcus*	*faecalis*		*lusitaniae*		*cloacae*
	freundii		*faecium*		*farmeri*	*Enterococcus*	*durans*
	koseri	*Escherichia*	*coli*	*Citrobacter*	*freundii*		*faecalis*
	xerosis	*Gardnerella*	*vaginalis*		*koserii*		*faecium*
	yungae	*Hafnia*	*alvei*		*braaki*		*gallinarum*
Corinebacterium	*jeikeium*	*Klebsiella*	*ornithinolytica*		*yungae*	*Klebsiella*	*ornithynolytica*
	urealyticum		*oxytoca*		*amalonaticus*		*oxytoca*
Enterobacter	*aerogenes*		*ozaenae*	*Corinabacterium*	*urealyticum*		*ozaenae*
	cancerogenus		*pneumoniae*		*xerosis*		*pneumoniae*
	cloacae	*Kluyvera*	*cryocrescens*		*striatum*	*Leclercia*	*adecarboxilata*
Enterococcus	*avium*	*Lattobacilli*	*spp*		*jeikeium (JK)*	*Morganella*	*morganii*

	durans	*Moraxella*	*morganii*	*Enterobacter*	*aerogenes*	*Pantoea*	*spp*
	faecalis	*Proteus*	*mirabilis*		*cloacae*	*Proteus*	*mirabilis*
	faecium		*vulgaris*	*Escherichia*	*coli*		*penneri*
	gallinarum	*Providencia*	*stuartii*	*Enterococcus*	*durans*		*vulgaris*
	raffinosus	*Roultella*	*planticola*		*gallinarum*	*Pseudomoas*	*aeruginosa*
Escherichia	*coli*	*Streptococcus*	*agalactiae*		*columbae*		*putida*
	fergusonii		*gallolyticus*		*faecalis*	*Serratia*	*fonticola*
Klebsiella	*oxytoca*		*mitis*		*faecium*		*marcescens*
	ozaenae		*hyointestinalis*		*avium*	*Staphylococcus*	*aureus*
	pneumoniae	*Staphilococcus*	*aureus*		*casseliflavus*		*haemolyticus*
Kokuria	*kristinae*		*carnosus*	*Morganella*	*morganii*		*hominis*
Lactococcus	*spp*		*epidermidis*	*Pantoea*	*spp*		*saprophyticus*
Lattobacilli	*spp*		*haemolyticus*	*Proteus*	*vulgaris*		*warneri*
Leclercia	*adecarboxylata*		*hominis*		*mirabilis*	*Streptococcus*	*agalactiae*
Morganella	*morganii*		*saprophiticus*	*Providencia*	*stuartii*		*gallolyticus*
Neisseria	*spp*	*Serratia*	*liquefaciens*		*rettgeri*		*viridans*
Pseudomonas	*aeruginosa*		*marcescens*	*Pseudomonas*	*aeruginosa*	*Stenotrophomonas*	*maltophilia*
	cepacea	*Stenotrophomonas*	*maltophylia*		*cepacea*		
	putida				*putida*		
Proteus	*mirabilis*			*Roultella*	*planticola*		
	penneri			*Serratia*	*fonticola*		
	vulgaris				*odorifera*		
Providencia	*rettgeri*				*liquefaciens*		
	stuartii				*marcescens*		
Pantoea	*spp*			*Sphingomonas*	*paucimobilis*		
Rodotorula	*species*			*Stenotrophomonas*	*maltophilia*		
Roultella	*planticola*			*Staphylococcus*	*aureus*		
Saccaromices	*cerevisiae*				*epidermidis*		
Serratia	*fonticola*				*hominis*		
	marcescens				*haemolyticus*		
Staphilococcus	*aureus*				*lentus*		
	capitis				*warneri*		

	epidermidis		*capitis*
	haemoliticus		*lugdunensis*
	hominis		*sciuri*
	lentus	*Streptococcus*	*gallolyticus*
	lugdunensis		*agalactiae*
	saprophiticus		
	simulans		
	warnieri		
Streptococcus	*agalactiae*		
	anginosus		
	gallolyticus		
	mutans		
Sphingomonas	*paucimobilis*		
